# Cellular and Molecular Mechanisms of REM Sleep Homeostatic Drive: A Plausible Component for Behavioral Plasticity

**DOI:** 10.3389/fncir.2017.00063

**Published:** 2017-09-14

**Authors:** Subimal Datta, Michael D. Oliver

**Affiliations:** ^1^Laboratory of Sleep and Cognitive Neuroscience, Graduate School of Medicine, Department of Anesthesiology, The University of Tennessee Knoxville, TN, United States; ^2^Department of Psychology, College of Arts and Sciences, The University of Tennessee Knoxville, TN, United States

**Keywords:** selective REM sleep restriction, intracellular signaling, rat, brainstem, U0126

## Abstract

Homeostatic regulation of REM sleep drive, as measured by an increase in the number of REM sleep transitions, plays a key role in neuronal and behavioral plasticity (i.e., learning and memory). Deficits in REM sleep homeostatic drive (RSHD) are implicated in the development of many neuropsychiatric disorders. Yet, the cellular and molecular mechanisms underlying this RSHD remain to be incomplete. To further our understanding of this mechanism, the current study was performed on freely moving rats to test a hypothesis that a positive interaction between extracellular-signal-regulated kinase 1 and 2 (ERK1/2) activity and brain-derived neurotrophic factor (BDNF) signaling in the pedunculopontine tegmentum (PPT) is a causal factor for the development of RSHD. Behavioral results of this study demonstrated that a short period (<90 min) of selective REM sleep restriction (RSR) exhibited a strong RSHD. Molecular analyses revealed that this increased RSHD increased phosphorylation and activation of ERK1/2 and BDNF expression in the PPT. Additionally, pharmacological results demonstrated that the application of the ERK1/2 activation inhibitor U0126 into the PPT prevented RSHD and suppressed BDNF expression in the PPT. These results, for the first time, suggest that the positive interaction between ERK1/2 and BDNF in the PPT is a casual factor for the development of RSHD. These findings provide a novel direction in understanding how RSHD-associated specific molecular changes can facilitate neuronal plasticity and memory processing.

## Introduction

A significant body of research, exploring the inner workings of learning and memory, has suggested that the mechanisms regulating REM sleep homeostatic drive (RSHD) are critical for the development, maturation and plasticity of the brain (Garcia-Rill, 1991; Datta, 2000; Garcia-Rill et al., 2008; Shaffery et al., 2012a,b; Dumoulin Bridi et al., 2015; Frank, 2015; Kocsis, 2016). For example, studies using a variety of training paradigms have shown that memory processing increases the homeostatic drive for sleep (Datta and O’Malley, 2013; Poe, 2017). Sleep homeostasis reflects a centrally mediated drive, which increases during sleep restriction and resolves during sleep recovery (Datta and Maclean, 2007; Datta et al., 2015). This homeostatic drive has also been linked to emotional regulation, memory consolidation, and cognitive function (Smith, 1995; Datta, 2006; Poe et al., 2010; Datta and O’Malley, 2013) and is absent in a number of neuropsychiatric disorders (Gottesmann and Gottesman, 2007; Khatami et al., 2008; Anderson and Bradley, 2013). Evidence suggests that the REM and non-REM sleep (NR) homeostatic regulatory processes are independent of each other (Vivaldi et al., 1994; Franken, 2002; Shea et al., 2008). The mechanisms for homeostatic regulation of NR are well documented, however, the mechanisms underlying homeostatic regulation of REM sleep remain incomplete (Faraguna et al., 2008; Thakkar et al., 2008; Datta et al., 2015; Bjorness et al., 2016).

Studies in rats have shown that the total amount of REM sleep during their normal sleep period is tightly regulated so that the amounts are relatively invariant (Shea et al., 2008; Datta et al., 2015; Barnes et al., 2017). Additionally, within a short period (2–3 h), during selective REM sleep restriction (RSR), there are progressively more attempts at transitioning into REM sleep, thus indicating a strong homeostatic drive for REM sleep (Shea et al., 2008; Datta et al., 2015; Barnes et al., 2017). Using short-term total sleep deprivation we have shown that RSHD develops during selective RSR but not during or after total sleep deprivation, suggesting that RSHD develops primarily during NR but not during wakefulness (Benington and Heller, 1994; Shea et al., 2008; Datta et al., 2015). Interestingly, this short-term selective RSR also increases the levels of brain-derived neurotrophic factor (BDNF) expression in the pedunculopontine tegmentum (PPT; one of the REM sleep generating areas), yet not in other sleep regulating areas of the brain (Datta et al., 2015).

It is evident that to expand our understanding of the mechanisms of memory processing and brain plasticity, it is critical to examine the cellular and molecular mechanisms responsible for the development of RSHD. Recently, it has been shown that the development of RSHD involves activation of PPT BDNF-TrkB receptors (Barnes et al., 2017). TrkB receptors may activate several intracellular signaling pathways, including extracellular-signal-regulated kinase 1 and 2 (ERK1/2) transduction pathway (Segal and Greenberg, 1996; Han and Holtzman, 2000; Huang and Reichardt, 2001; Ying et al., 2002; Kishino and Nakayama, 2003; Alonso et al., 2004; Lu et al., 2004; Mohajerani et al., 2007; Numakawa et al., 2010). Furthermore, ERK1/2 signaling may increase BDNF release in the brain in a positive feedback loop (Obata et al., 2003; Kelleher et al., 2004; Klann and Dever, 2004). Therefore, in this study, we aim to test the hypothesis that increased ERK1/2 signaling in the PPT is critical for BDNF release and the development of RSHD.

## Materials and Methods

### Subjects and Housing

Experiments were performed on 34 adult male Wistar rats (Charles River, Wilmington, MA, USA) weighing between 250 g and 350 g. The rats were housed singly at 24°C under a 12 h light/dark cycle (lights on from 6:00 a.m. to 6:00 p.m.) with free access to food and water. To reduce additional stress that might be imposed by experimental handling, animals underwent a period of habituation during which they were gently handled daily for 15–20 min between 9:00 a.m. and 10:00 a.m. All procedures were performed in accordance with the NIH Guide for the Care and Use of Laboratory Animals and were approved by the University of Tennessee Animal Care Committee (Protocol Number: 2311-1214-UTK). All experiments are conducted in compliance with the ARRIVE guidelines (Kilkenny et al., 2010). On the basis of ensuring the validity of experimental results, efforts were made to reduce the number of animals used in our experiments and to minimize any possible suffering by the animals.

### Surgical Procedures

All surgical procedures were performed with rats secured in a stereotaxic frame under isoflurane anesthesia (2%–3% isoflurane and 1.4 L/min 100% O_2_) and pretreated with an analgesic (buprenorphine; 0.05 mg/kg, I.M.; Cerilliant, Round Rock, TX, USA). During surgery, core body temperature was maintained at 37°C ± 1°C with a thermostatic heating pad. Upon completion, animals were administered saline (5 cc, s.c.) to prevent dehydration and buprenorphine (0.02 mg/kg, I.M.) to control potential post-operative pain.

### Implantation of Electroencephalogram and Electromyogram Recording Electrodes and Guide Tubes for the Localized Microinjections of Drugs into the PPT

To record behavioral states of vigilance, cortical electroencephalogram (EEG), dorsal neck muscle electromyogram (EMG), and hippocampal EEG (theta wave) recording electrodes were chronically implanted, as described previously (Datta et al., 2002). Stainless steel guide tubes (26-gauge) with equal length stylettes were stereotaxically implanted bilaterally 2 mm above the PPT (in relation to stereotaxic “0”: anterior, 1.25; lateral, 2.0; horizontal, 3.0; Paxinos and Watson, 1997), as described previously (Datta, 2002; Datta et al., 2011). All electrodes and guide tubes were secured to the skull with dental acrylic and the electrodes were crimped to mini-connector pins and brought together in a plastic connector.

### Drug and Vehicle Control Microinjections

The drug used in this study was a specific inhibitor of ERK1/2 activation, 1,4-Diamino-2,3-dicyano-1,4-bis[2-a minophenylthio] butadiene (U0126; Mol. Wt. 380.48), purchased from Santa Cruz Biotechnology, Inc. (Dallas, TX, USA). The U0126 was initially dissolved in dimethyl sulfoxide (DMSO) and then diluted in 0.9% saline at four concentrations (0.5, 1.0, 2.0, and 5.0 nmol/100 nl). For vehicle control microinjection, a solution was prepared by mixing 20% volume of DMSO in 0.9% saline. Vehicle control and drug solutions were freshly prepared under sterile conditions immediately before each use. The selection of this drug was based on its selective inhibitory effect on intracellular MAP kinase kinase activation (Favata et al., 1998; Namura et al., 2001). Commercially available U0126 is suitable for microinjection studies because it is cell-permeable and has reversible effects. It has also been used successfully in past microinjection studies (Roberson et al., 1999; Obata et al., 2003; Apergis-Schoute et al., 2005; Schafe et al., 2005; Herry et al., 2006; Huang and Lin, 2006; Fischer et al., 2007; Whitfield et al., 2011; Kang et al., 2013).

### Localized Microinjections of U0126 and Vehicle Control into the PPT

The microinjection system consisted of a 32-gauge stainless steel injector cannula with a 26-gauge collar that extended 2.0 mm beyond the implanted guide tube. The collar was connected to a 1.0 μl motor-driven Hamilton microsyringe with PE 20 tubing. While the animal was connected to the recording system, the stylettes were removed and an injector filled with either vehicle control (100 nl volume) or one of the three concentrations of U0126 (0.5, 1.0, and 2.0 nmol/100 nl) was introduced through one of the bilateral guide tubes for injection. This procedure was then repeated in the other guide tube. One minute after the insertion of the injector cannula, 100 nl of vehicle control or one of the three concentrations of U0126 was bilaterally microinjected over a 60 s period (Datta and Desarnaud, 2010; Datta et al., 2011). The injector cannulae were gently withdrawn 2 min after the injections, and the stylettes were reinserted into the guide tubes. During the microinjections, animals were free to move around the cage with the cannulae in place. Each rat received a total of two microinjections (100 nl each, one in the right and one in the left PPT) in a single experimental recording session. Based on our earlier publications and experiences with microinjections of pharmacologically active substances in the PPT, we are confident that the significant pharmacological effects observed after microinjection of U0126 occurred only within the PPT (Datta et al., 2011).

### Sleep-Wake Recording and Analysis

The preamplifier connected to each rat’s head amplified the cortical EEG, hippocampal EEG and EMG signals by 100×. The commutator received these signals, which were then conditioned by an analog filter. EEG signals were sampled at 1 kHz and band-pass filtered between 0.5 Hz and 100 Hz. EMG signals were sampled at 2 kHz and band-pass filtered between 10 Hz and 200 Hz. In addition to these physiological signals, animals’ sleep-wake behavioral activities were monitored using a video camera attached above the recording cages. This synchronously recorded behavioral and physiological data was imaged and recorded using Sirenia^®^ Acquisition software (Pinnacle Technology Inc., Lawrence, KS, USA). Using Sirenia^®^ Sleep Pro software (Pinnacle Technology Inc., Lawrence, KS, USA), the recorded data was visually scored by an investigator blinded to the treatment conditions. Three behavioral states were distinguished: wake (W), non-REM and REM sleep. The physiological criteria for the identification of these wake-sleep states are described in detail in earlier publications (Datta et al., 2002, 2004). In the present study, the behavioral states of W, non-REM and REM sleep were scored in successive 5-s epochs.

### Selective REM Sleep Restriction

To study the cellular and molecular mechanisms for the development of RSHD, in this study we are using a selective RSR protocol. For the purpose of RSR, the beginning of each REM sleep episode was identified by observation of ongoing physiological signs (cortical EEG, hippocampal EEG and EMG) and video of behavioral activity. From the room adjacent to the rat, where the experimenter is observing the physiological signs and videos of animal behavior, the experimenter pressed a mechanical lever within 2–3 s of REM sleep onset, the animal’s head was gently lifted (between 1.0″ and 1.5″), and the animal was awakened. One of the most important advantages of this RSR method is that this method successfully eliminates >75% of REM sleep without significantly reducing SWS and another advantage of this method is that it can induce homeostatic drive for REM sleep within a very short period of time (Datta et al., 2004, 2015). This “head-lifting method” for RSR requires a small, spring action mechanical lever, three pulleys with equal wheel diameter, and a flexible, lightweight wire. The first pulley is positioned on the ceiling at a 90° angle, 3 ft above the commutator. The second pulley is located in the next room, positioned at the same height as the first pulley. The second pulley hangs from the ceiling above the computer monitor used for observing polygraphic signs. The third pulley is on the table with the computer monitor. The mechanical lever is fixed to the table about 6″ in front of the monitor. One end of the wire is tied to the commutator and the other end goes up, passes through the first, second and third pulleys, and then is finally tied to the mechanical lever. A relaxed spring keeps the mechanical lever in the up position. As needed, manually applied incremental downward pressure on the lever handle produces incremental lever action to raise the rat’s head by up to 2″ and terminate REM sleep.

### Adaptation Recording Session

After a post-surgical recovery period of 3–7 days, rats were habituated to the experimenter and free-moving polygraphic recording conditions for 7–10 days. Adaptation recording sessions were performed between 10:00 a.m. and 1:00 p.m., when rats are normally sleeping. These 3-h sessions allowed for electrode testing and daily observation of variations in wake-sleep activity. During this time, rats were placed in the recording cage (Pinnacle 8238: 12″ diameter × 12″ tall; Pinnacle Technology Inc., Lawrence, KS, USA) with a food hopper and a water bottle. A multichannel preamplifier (customized from Pinnacle Technology’s 8400 four-channel EEG/EMG system) was secured to the plastic connector on the head of each rat. A flexible recording cable connected each preamplifier to an electric swivel (Pinnacle 8409 rat commutator) fixed above each cage, allowing rats complete freedom of movement. The last day of adaptation recording sessions were as determined when, for three consecutive days, day-to-day variation in the percentage of REM sleep was less than 5% of the total amount of REM sleep for three consecutive days. This session was used as each rat’s baseline recording. During the periods of recovery and habituation, all rats were housed under the same 12/12 h light/dark cycle with free access to food and water.

### Experimental Design

On the day after the last adaptation recording session, experimental recording sessions began during which each animal was connected to the sleep-wake recording system at 9:55 a.m. The 30 rats were randomly divided into five groups. Group 1 (*n* = 6 rats): animals received bilateral microinjections of vehicle control (100 nl each in the right and left PPT) at 10:00 a.m. while being recorded for a 3-h session (between 10:00 a.m. and 1:00 p.m.) of undisturbed wake-sleep activity (hereafter, Group 1 is labeled as vehicle control and REM sleep control; “VC + RSC”). Group 2 (*n* = 6 rats): similar to Group 1, except that for Group 2 animals, from 10:00 a.m. to 1:00 p.m., REM sleep episodes were selectively terminated at the beginning (within 3–5 s) of each episode, as above (hereafter, group 2 is labeled as vehicle control and selective REM sleep deprivation; “VC + RSD”). Group 3 (*n* = 6 rats): similar to Group 2, except that Group 3 animals were microinjected with a 0.5 nmol concentration of U0126 (hereafter, group 3 is labeled as 0.5 nmol U0126 and selective REM sleep deprivation; “0.5 nmol U0126 + RSD”). Group 4 (*n* = 6 rats): similar to Group 3, except that Group 4 animals were microinjected with a 1.0 nmol concentration of U0126 (hereafter, group 4 is labeled as 1.0 nmol U0126 and selective REM sleep deprivation; “1.0 nmol U0126 + RSD”). Group 5 (*n* = 6 rats): similar to Group 4, except that Group 5 animals were microinjected with a 2.0 nmol concentration of U0126 (hereafter, group 5 is labeled as 2.0 nmol U0126 and selective REM sleep deprivation; “2.0 nmol U0126 + RSD”). Immediately after the end of the 3-h recording session the rats were quickly euthanized in order to quantify BDNF, pERK1/2 expression, and ERK1/2 activity in the PPT. The 3-h post-injection wake-sleep values of the drug-treated groups were then compared with those of the vehicle-control-treated groups to examine the various concentration-dependent effects of U0126 application into the PPT on selective REM sleep deprivation-induced increased homeostatic drive for REM sleep.

### Tissue Collection and Quantification of BDNF and Assessment of ERK1/2 Phosphorylation and Activity

**Figure 1:**
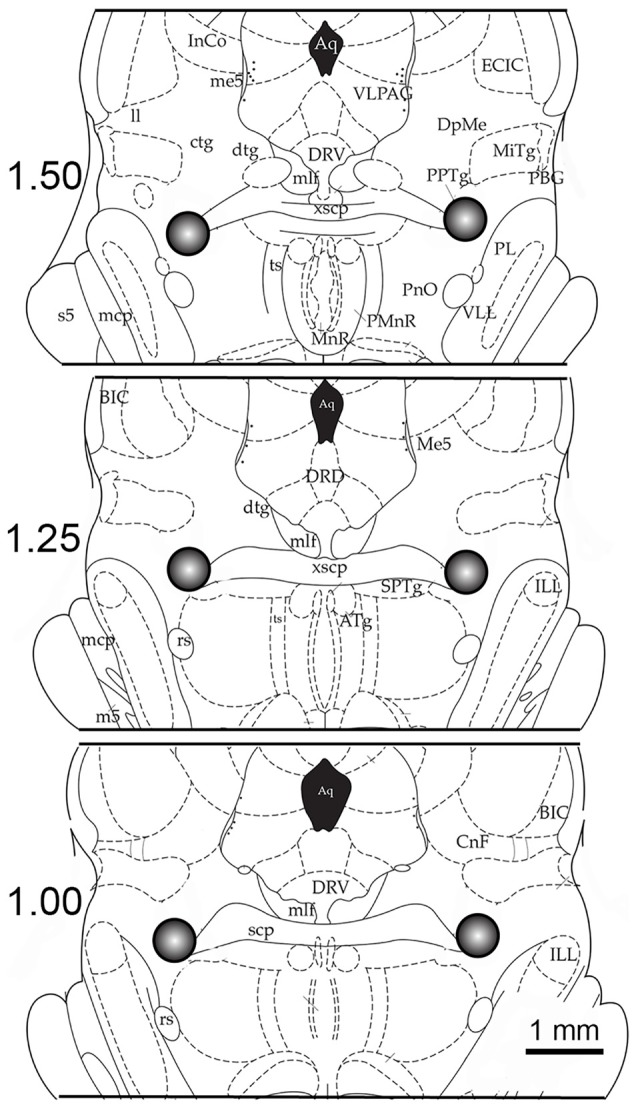
Example of the anatomical location of microinjection sites and tissue punch areas. Schematic coronal sections (250-micron thickness) through the brainstem are illustrated at levels 1.50 mm, 1.25 mm and 1.00 mm anterior (in relation to stereotaxic zero; Paxinos and Watson, 1997). The 0.50 mm cubic area that was dissected out, which includes the pedunculopontine tegmentum (PPT), is shown in the gray circle. The centers of these tissue punches were also the location of the microinjection site. Abbreviations: Aq, aqueduct; ATg, anterior tegmental nucleus; BIC, nucleus brachium inferior colliculus; CnF, cuneiform nucleus; ctg, central tegmental tract; dtg, dorsal tegmental bundle; DpMe, deep mesencephalic; DRD, dorsal raphe dorsal subnucleus; DRV, dorsal raphe ventral subnucleus; ECIC, external inferior colliculus; ILL, intermedial nucleus lateral lemniscus; InCo, intercollicular nucleus; mlf, medial longitudinal fasciculus; ll, lateral lemniscus; mcp, midcerebellar peduncle; m5, motor root trigeminal nucleus; Me5, mesencephalic 5 nucleus; MiTg, microcellular tegmental nucleus; PL, paralemniscal nucleus; PnO, pontine reticular nucleus; PPTg, pedunculopontine tegmental nucleus; rs, rubrospinal tract; scp, superior cerebellar peduncle; s5, sensory root trigeminal nucleus; ts, tectospinal tract; VLL, ventral nucleus lateral lemniscus; VLPAG, ventral periaqueductal gray; xscp, decussation of the superior cerebellar peduncle.

Immediately after the end of the experimental recording session (at 1:00 p.m.), rats were euthanized with carbon dioxide, and their brains were removed and frozen using dry ice. To minimize possible variations, due to differences in sleep-wake state at time of death, all animals were awakened and kept awake for 1 min before they were euthanized. All rats were euthanized at a fixed time of day to rule out any diurnal factors contributing to the different levels of ERK1/2 phosphorylation and activity. The frozen brains were cut in the transverse plane in 250 μm thick sections with a Vibratome (series 3000; Technical Products International, Inc., St. Louis, MO, USA). The PPT was dissected, under a dissecting microscope, on an ice-chilled Petri dish, as described earlier (Datta and Desarnaud, 2010). The PPT tissue collection region was: antero-posterior, between 0.75 mm and 1.75 mm anterior to the stereotaxic zero; medial-lateral, between 1.75 mm and 2.25 mm lateral to the midline (see Figure 1). The tissue punches (0.5 mm diameter tissue punch tool) from the PPT region, from both the left and right sides of the brain of each individual rat, was processed immediately for the quantification of BDNF, ERK1/2 phosphorylation and ERK1/2 activity. The amount of BDNF (BDNF/mg total protein) was determined using a standard ELISA technique, as described earlier (Datta et al., 2009, 2015). Similarly, the levels of ERK1/2 phosphorylation and activation were assessed using western blotting techniques, as described in our earlier publications (Desarnaud et al., 2011).

### Statistical Analysis

To determine the effects of intra-PPT microinjections of U0126 on changes in RSHD, the physiological measures of the rats were analyzed to calculate the following dependent variables, which were quantified for each recording session: (1) percentage of recording time spent in W, non-REM and REM sleep; and (2) total number of REM sleep episodes. The effects of the five different treatments (VC + RSC; VC + RSD; 0.5 nmol U0126 + RSD; 1.0 nmol U0126 + RSD; and 2.0 nmol U0126 + RSD) on the percentages of W, non-REM, REM sleep and total number of REM sleep episodes were statistically analyzed using one-way ANOVAs followed by *post hoc* tests (Bonferroni’s multiple comparisons test). Similarly, one-factor ANOVAs and *post hoc* tests (Bonferroni’s multiple comparisons test) were used to compare the levels of ERK1/2 phosphorylation and activity and amount of BDNF in the PPT of five different treatment groups (VC + RSC; VC + RSD; 0.5 nmol U0126 + RSD; 1.0 nmol U0126 + RSD; and 2.0 nmol U0126 + RSD). To assess the predictive relationship between the levels of PPT ERK1/2 activity and increased RSHD, during the 3 h period of the selective REM sleep deprivation (between 10:00 a.m. and 1:00 p.m.), a regression analysis was performed between PPT ERK1/2 activity and total number of REM sleep episodes. Prior to statistical analysis, group data was subjected to normality testing confirming that normality assumptions were met. The threshold for significance was *p* < 0.05. All statistical analyses were performed using Graphpad Prism statistical software (v5.0; Graphpad Software, La Jolla, CA, USA).

## Results

**Figure 2:**
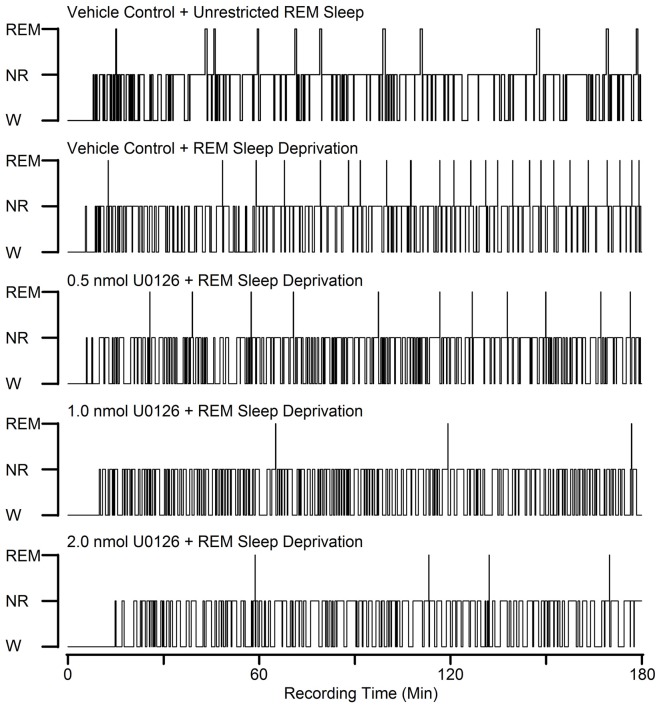
Examples to show the effects of bilateral microinjections of different doses of U0126 into the PPT on sleep-wake architecture during selective REM sleep deprivation. These five hypnograms from five different rats plotted as step histograms plot the occurrence and duration of polygraphically defined wakefulness (W), non-REM sleep (NR), and REM sleep after vehicle control and three different doses of U0126. All microinjections were made at 10:00 A.M. and were followed by 3 h recording session. Note increased REM sleep homeostatic drive (RSHD; progressively increased attempt to REM sleep) in animals treated with vehicle control and selective REM sleep deprivation compared to the vehicle control and unrestricted REM sleep. Also note that the numbers of REM sleep episodes in the U0126-treated rats are reduced compared to the vehicle control and selective REM sleep deprived rat.

During the baseline recording session (between 10:00 a.m. and 1:00 p.m.), there were no significant differences (one-factor ANOVAs) among the five treatment groups (VC + RSC, VC + RSD, 0.5 nmol U0126 + RSD, 1.0 nmol U0126 + RSD, and 2.0 nmol U0126 + RSD) in terms of total percentage of time spent in W, NR and REM sleep and total number of REM sleep episodes (Table 1). Thus, during the identical circadian phase under final baseline recording conditions, the groups were equal in terms of wake-sleep variables. Additionally, in the vehicle control group of rats (VC + RSC), the microinjection recording session was comparable to the final baseline recording session in terms of time spent in REM sleep (11.0 ± 0.82% vs. 10.0 ± 0.56%) and number of REM sleep episodes (9.3 ± 0.76 vs. 7.8 ± 0.79). These results suggest that microinjection of vehicle control did not significantly change RSHD. During experimental recording session (between 10:00 a.m. and 1:00 p.m.), during selective REM sleep deprivation immediately after bilateral microinjection of microinjection of control vehicle, five different groups of rats exhibited five different patterns of sleep-wake architectures (Figure 2). These results suggest that microinjection of U0126 and selective REM sleep deprivation have changed sleep-wake architecture and RSHD. Additionally, to determine the effects of U0126 alone, we microinjected 5.0 nmol U0126 bilaterally into the PPT of four rats, followed by a 6-h recording of undisturbed sleep-wake activity. During the first 3-h of the experimental recording period, total percentages of time spent in wakefulness (mean ± SEM: 55.6 ± 6.9) were higher and total percentages of time spent in NR (38.9 ± 3.6), REM sleep (6.2 ± 1.2), and number of REM sleep episodes (5.5 ± 0.41) were lower than those in the control (VC + RSC) group of rats. These results show that microinjection of U0126 into the PPT has a tendency to reduce sleep, but it does not totally eliminate sleep. However, during the last 3-h of recordings, it was revealed that the sleep-wake values were comparable to the baseline level and there was no indication of a REM sleep rebound. These results indicated that U0126 was successful at RSD-induced RSHD without any major effects on spontaneously occurring REM sleep episodes.

**Table 1:** The total percentages (mean ± SEM) of time spent in wakefulness, non-REM sleep and REM sleep and total number of REM sleep episodes in five different groups of rats (*n* = 6 rats/group) in the 3-h baseline sleep-wake recording session (undisturbed recordings from 10:00 A.M. to 1:00 P.M.).

Group	VC + RSC	VC + RSD	0.5 nmol U0126 + RSD	1.0 nmol U0126 + RSD	2.0 nmol U0126 + RSD	Statistics
Wakefulness (%)	37.5 ± 2.6	34.1 ± 1.8	35.8 ± 1.5	34.0 ± 2.6	35.2 ± 3.1	*F*_(4,25)_ = 0.29; *P* = 0.88
Non-REM sleep (%)	50.5 ± 3.0	55.0 ± 2.3	52.2 ± 2.1	54.0 ± 2.6	52.8 ± 3.4	*F*_(4,25)_ = 0.18; *P* = 0.95
REM sleep (%)	11.0 ± 0.82	10.9 ± 1.2	12.0 ± 0.93	12.0 ± 0.72	12.0 ± 0.89	*F*_(4,25)_ = 0.34; *P* = 0.85
Number of REM sleep	9.3 ± 0.76	9.8 ± 1.2	11.0 ± 0.71	9.5 ± 0.76	10.0 ± 1.2	*F*_(4,25)_ = 0.29; *P* = 0.88
episodes						

*Abbreviations: RSC, REM sleep control; RSD, REM sleep deprivation; VC, vehicle control*.

### Application of U0126 into the PPT Prevented Selective RSD-Induced RSHD

**Figure 3:**
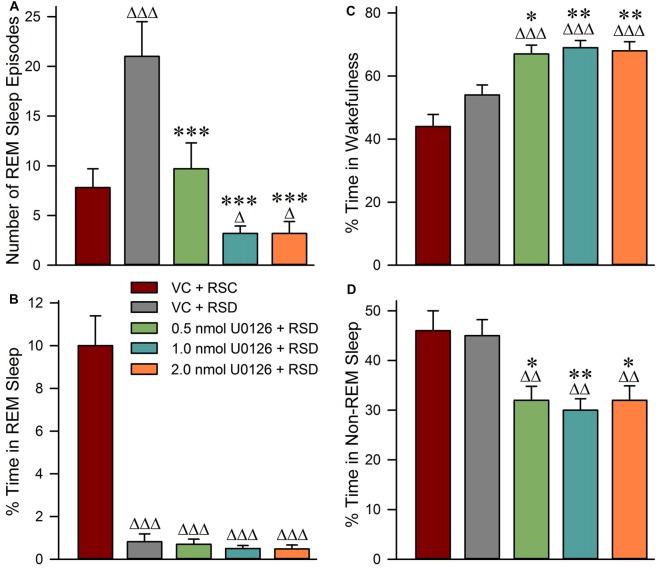
Application of U0126 into the PPT suppresses selective REM sleep deprivation-induced REM sleep homeostatic pressure. This effect was observed during the 3-h sleep-wake recording session that immediately followed bilateral microinjections of U0126. **(A)** Bars represent number (mean ± SE) of REM sleep episodes. **(B)** Bars represent total percentages of time (mean ± SE) spent in REM sleep. **(C)** Bars represent percentages of time spent in wakefulness. **(D)** Bars represent percentages of time spent in NR. *Post hoc* tests (Bonferroni’s multiple comparisons test): triangle represents the comparison with vehicle control + REM sleep control (VC + RSC) and asterisk represents the comparison with vehicle control + selective REM sleep deprivation (VC + RSD). ∆*p* < 0.05; ∆∆∆ or ***p < 0.001.

Selective RSD-induced changes in RSHD following bilateral microinjections of vehicle control and the different concentrations of U0126 into the PPT are summarized in Figure 3. One-way ANOVAs indicated a significant treatment effect in both the total number of episodes (*F*_(4,25)_ = 66; *p* < 0.001) and total percentages of time spent (*F*_(4,25)_ = 261; *p* < 0.001) in REM sleep among the five different treatment groups. The results of *post hoc* analysis (Bonferroni’s multiple comparisons tests) of the number of REM sleep episodes during the experimental recording session are presented in Figure 3A. Individual *post hoc* analysis indicated that, in the “VC + RSD” group, the numbers of REM sleep episodes were significantly higher (169.23% higher; *t* = 10; *df* = 25; *p* < 0.001) compared with an identical circadian time period of the “VC + RSC” group. This increase of REM sleep episodes in the “VC + RSD” group compared with the “VC + RSC” group suggests that during this selective REM sleep deprivation there were progressively more frequent attempts at transitions into REM sleep; an indication of a strong RSHD. Similar *post hoc* analysis indicated that the number of REM sleep episodes were significantly less in the “0.5 nmol U0126 + RSD” (53.81% less; *t* = 8.9; *df* = 25; *p* < 0.001), “1.0 nmol U0126 + RSD” (84.76% less; *t* = 14; *df* = 25; *p* < 0.00), and “2.0 nmol U0126 + RSD” (84.76% less; *t* = 14; *df* = 25; *p* < 0.00) treatment groups, compared with “VC + RSD” group (Figure 3A). These results suggest that the application of U0126 into the PPT successfully prevented selective RSD-induced RSHD in a dose dependent manner. The results of *post hoc* analysis (Bonferroni’s multiple comparisons tests) of the total percentages of time spent in REM sleep during the experimental recording session are presented in Figure 3B. Individual *post hoc* analysis indicated that the total percentages of time spent in REM sleep were significantly less in the “VC + RSD” (91.8% less; *t* = 25.0; *df* = 25; *p* < 0.001), “0.5 nmol U0126 + RSD” (93.0% less; *t* = 25.0; *df* = 25; *p* < 0.001), “1.0 nmol U0126 + RSD” (95.0% less; *t* = 26.0; *df* = 25; *p* < 0.001), and “2.0 nmol U0126 + RSD” (95.2% less; *t* = 26.0; *df* = 25; *p* < 0.001) treatment groups, compared with “VC + RSC” group (Figure 3B). These results demonstrated that this short-term selective RSD protocol effectively eliminated more than 91% of total REM sleep.

### Application of U0126 into the PPT Increased Wakefulness and Decreased Non-REM Sleep during Selective RSD

Selective RSD-induced changes in wakefulness and NR during experimental recording sessions are summarized in Figure 3. One-way ANOVAs indicated a significant treatment effect in percentages of time spent in both wakefulness (*F*_(4,25)_ = 18; *p* < 0.001) and NR (*F*_(4,25)_ = 9; *p* < 0.001) among the five different treatment groups. The results of *post hoc* analysis (Bonferroni’s multiple comparisons tests) of the percentages of time spent in wakefulness during the experimental recording session are presented in Figure 3C. *Post hoc* analysis revealed that the total percentage of time spent in wakefulness in the “VC + RSD” group was not significantly different than in “VC + RSC” group. This result demonstrated that this short-term selective RSD protocol did not increase wakefulness. Similar comparisons revealed that the total percentages of time spent in wakefulness were significantly higher in the “0.5 nmol U0126 + RSD” (24.07% higher; *t* = 3.6; *df* = 25; *p* < 0.05), “1.0 nmol U0126 + RSD” (27.78% higher; *t* = 4.2; *df* = 25; *p* < 0.01), and “2.0 nmol U0126 + RSD” (25.93% higher; *t* = 3.8; *df* = 25; *p* < 0.01) treatment groups, compared with “VC + RSD” group (Figure 3C). These results suggested that microinjections of U0126 into the PPT increased the total percentage of time spent in wakefulness during the 3 h period of selective RSD.

The results of *post hoc* analysis of the percentage of time spent in NR during the experimental recording session are presented in Figure 3D. *Post hoc* analysis revealed that the total percentage of time spent in NR in the “VC + RSD” group was not significantly different than in “VC + RSC” group. This result demonstrated that this short-term selective RSD protocol did not decrease NR. However, the total percentage of time spent in NR were significantly less in the “0.5 nmol U0126 + RSD” (28.89% less; *t* = 3.5; *df* = 25; *p* < 0.05), “1.0 nmol U0126 + RSD” (33.33% less; *t* = 4.1; *df* = 25; *p* < 0.01), and “2.0 nmol U0126 + RSD” (28.89% less; *t* = 3.7; *df* = 25; *p* < 0.05) treatment groups, compared with “VC + RSD” group (Figure 3D). These results suggested that microinjections of U0126 into the PPT decreased the total percentages of time spent in NR during the 3 h period of selective RSD.

### Microinjections of U0126 into the PPT Suppressed Selective RSD-Induced Increased Phosphorylation and Activation of ERK1/2 and Increased BDNF in the PPT

**Figure 4:**
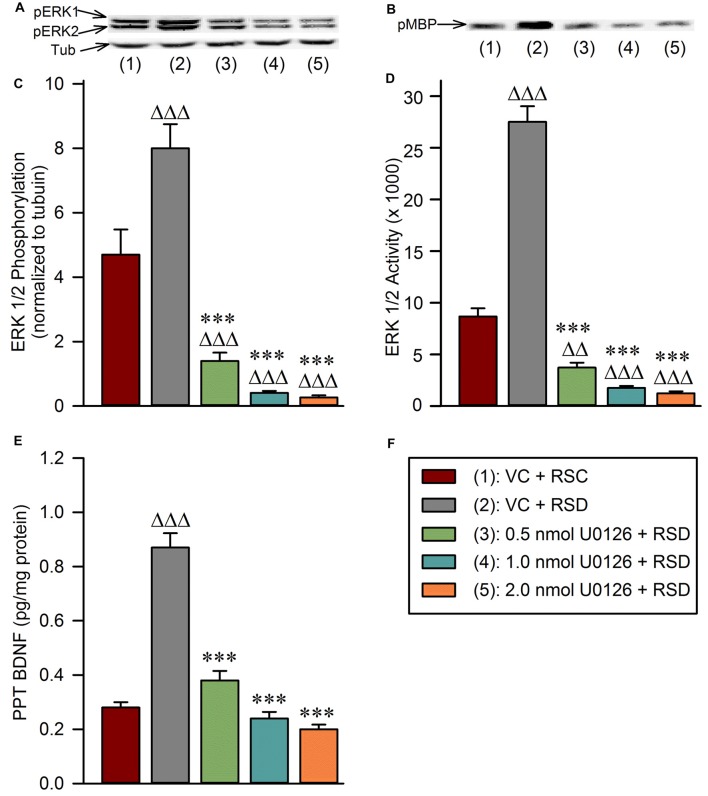
Local application of U0126 into the PPT prevents selective REM sleep deprivation-induced increases in BDNF protein expression and extracellular-signal-regulated kinase 1 and 2 (ERK1/2) phosphorylation and activation in the PPT. **(A)** Representative western blots of pERK1/2 and α-tubulin in the PPT of animals in the following groups: (1) vehicle control injected unrestricted REM sleep (VC + RSC); (2) vehicle control injected selective REM sleep deprived (VC + RSD); (3) 0.5 nmol U0126 injected selective REM sleep deprived (0.5 nmol U0126 + RSD); (4) 1.0 nmol U0126 injected selective REM sleep deprived (1.0 nmol U0126 + RSD); and (5) 2.0 nmol U0126 injected selective REM sleep deprived (2.0 nmol U0126 + RSD). **(B)** Representative western blots of pMBP in the PPT of: (1) VC + RSC; (2) VC + RSD; (3) 0.5 nmol U0126 + RSD; (4) 1.0 nmol U0126 + RSD; and (5) 2.0 nmol U0126 + RSD. **(C)** Bars represent levels (mean ± SE) of phosphorylated ERK1/2 in the PPT of: (1) VC + RSC; (2) VC + RSD; (3) 0.5 nmol U0126 + RSD; (4) 1.0 nmol U0126 + RSD; and (5) 2.0 nmol U0126 + RSD. All analyses of pERK1/2 expression are normalized against α-tubulin. **(D)** Bars represent densitometric measurements (mean ± SE) from western blots of phosphorylated myelin basic protein (pMBP) levels in the PPT of: (1) VC + RSC; (2) VC + RSD; (3) 0.5 nmol U0126 + RSD; (4) 1.0 nmol U0126 + RSD; and (5) 2.0 nmol U0126 + RSD. **(E)** Bars represent amounts (mean ± SE) of BDNF in the PPT of: (1) VC + RSC; (2) VC + RSD; (3) 0.5 nmol U0126 + RSD; (4) 1.0 nmol U0126 + RSD; and (5) 2.0 nmol U0126 + RSD. **(F)** Key. *Post hoc* test (Bonferroni’s multiple comparisons test): triangle represents the comparison with vehicle control + REM sleep control (VC + RSC) and asterisk represents the comparison with vehicle control + selective REM sleep deprivation (VC + RSD). ∆∆∆ or ***p < 0.001.

One-way ANOVAs revealed significant treatment effects in the PPT levels of pERK1/2 (*F*_(4,25)_ = 45; *p* < 0.001; Figure 4A), ERK1/2 activity (*F*_(4,25)_ = 190; *p* < 0.001; Figure 4B), and BDNF protein (*F*_(4,25)_ = 72; *p* < 0.001) among the five different treatment groups (Figure 4F). The results of *post hoc* analyses (Bonferroni’s multiple comparisons tests) of the PPT levels of pERK1/2 are presented in Figure 4C. *Post hoc* analysis revealed that the level of pERK1/2 in the “VC + RSD” group was significantly higher (70.21% more; *t* = 4.6; *df* = 25; *p* < 0.001) than in “VC + RSC” group. On the contrary, PPT levels of pERK1/2 were significantly less in the “0.5 nmol U0126 + RSD” (82.5% less; *t* = 9.3; *df* = 25; *p* < 0.001), “1.0 nmol U0126 + RSD” (94.88% less; *t* = 11.0; *df* = 25; *p* < 0.001), and “2.0 nmol U0126 + RSD” (96.63% less; *t* = 11.0; *df* = 25; *p* < 0.001) treatment groups, compared with “VC + RSD” group (Figure 4C). Like the levels of pERK1/2, the levels of ERK1/2 activity in the PPT in the “VC + RSD” group were significantly higher (216.98% more; *t* = 17.0; *df* = 25; *p* < 0.001) than in “VC + RSC” group (Figure 4D). *Post hoc* analysis also revealed that the PPT levels of ERK1/2 activity were significantly less in the “0.5 nmol U0126 + RSD” (86.46% less; *t* = 21.0; *df* = 25; *p* < 0.001), “1.0 nmol U0126 + RSD” (93.63% less; *t* = 23.0; *df* = 25; *p* < 0.001), and “2.0 nmol U0126 + RSD” (95.47% less; *t* = 11.0; *df* = 25; *p* < 0.001) treatment groups, compared with “VC + RSD” group (Figure 4D). Collectively, these results demonstrated that this short-term selective RSD increased the PPT levels of both ERK1/2 phosphorylation and ERK1/2 activity. These results also suggested that the local microinjections of U0126 into the PPT prevented selective RSD-induced increased phosphorylation and activation of ERK1/2 in the PPT.

The results of *post hoc* analyses of the PPT levels of BDNF protein are presented in Figure 4E. The levels of BDNF in the PPT in the “VC + RSD” group were significantly higher (210.71% more; *t* = 13.0; *df* = 25; *p* < 0.001) than in “VC + RSC” group (Figure 4E). *Post hoc* analyses also revealed that the levels of BDNF in the PPT were significantly less in the “0.5 nmol U0126 + RSD” (56.32% less; *t* = 11.0; *df* = 25; *p* < 0.001), “1.0 nmol U0126 + RSD” (72.41% less; *t* = 14.0; *df* = 25; *p* < 0.001), and “2.0 nmol U0126 + RSD” (77.01% less; *t* = 15.0; *df* = 25; *p* < 0.001) treatment groups, compared with “VC + RSD” group (Figure 4E). These results indicate that this short-term selective RSD protocol increased levels of BDNF in the PPT, and application of U0126 into the PPT suppressed this selective RSD-induced PPT BDNF increase.

### Relationship between RSD-Induced Changes in the Amount of BDNF and ERK1/2 Activity in the PPT and REM Sleep Pressure

**Figure 5:**
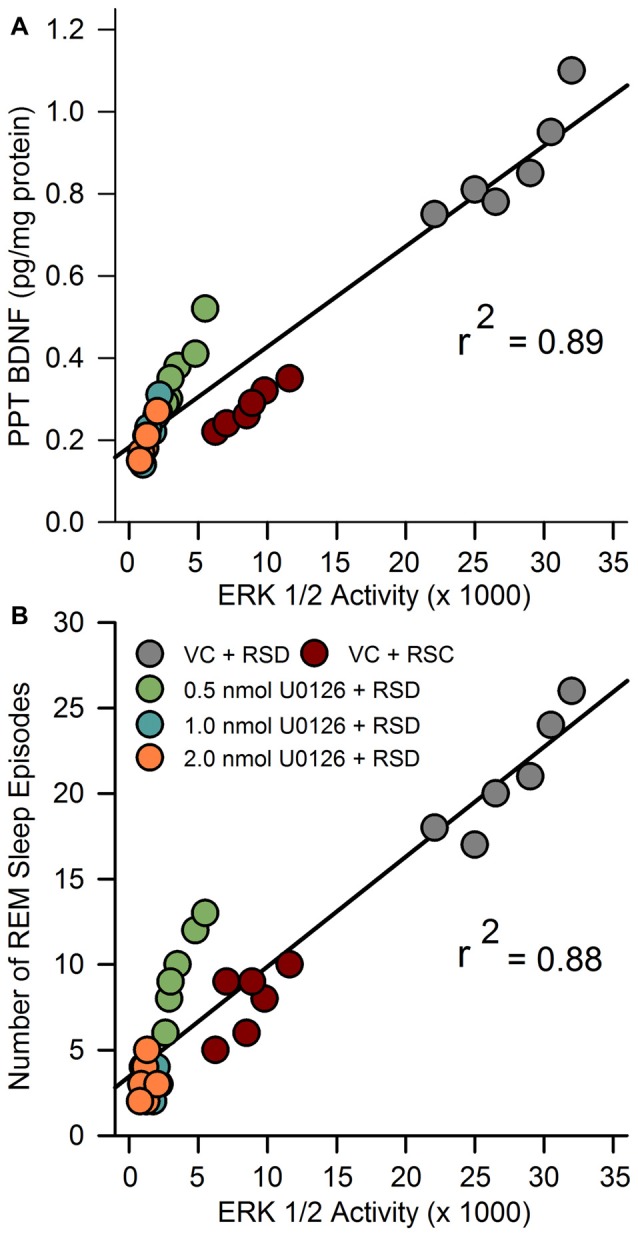
Molecular correlates of RSHD. **(A)** The relationship between selective REM sleep deprivation (RSD)-induced changes in amount of BDNF protein and ERK1/2 activity in the PPT of rats treated with vehicle control and different concentrations of U0126. Plot of linear regression best fit (solid line) showed a statistically significant positive slope (*R*^2^ = 0.89; *p* < 0.001) between ERK1/2 activity and individual animals’ amount of BDNF in the PPT. **(B)** Relationships between selective RSD-induced changes in RSHD, as measured by number of REM sleep episodes, and changes in ERK1/2 activity in the PPT. Plots of linear regression best fit (solid line; Pearson product-moment correlation) revealed a significant relationship between number of REM sleep episodes and level of ERK1/2 activity in the PPT (*R*^2^ = 0.88; *p* < 0.001).

In our earlier study, we suggested that during selective RSD, increased BDNF in the PPT might be involved in the development of REM sleep pressure (Datta et al., 2015). Currently, we have documented that selective RSD increased ERK1/2 activity in the PPT. Thus, to identify a possible relationship between RSD-induced changes in BDNF and ERK1/2 activity linear regression analyses were performed between individual animals’ BDNF and ERK1/2 activity levels in the PPT. Linear regression analysis revealed a significant positive relationship (*F*_(1,28)_ = 250.3; *R*^2^ = 0.89; *p* < 0.001) between ERK1/2 activity and BDNF level in the PPT (Figure 5A).

We have also documented that the microinjections of U0126 into the PPT decreased selective RSD-induced increases in RSHD and ERK1/2 activity in the PPT. Thus, we expected to see a significant relationship (linear regression) between individual animals’ levels of ERK1/2 activity in the PPT and total number of REM sleep episodes. The results of this regression analysis revealed a significant positive relationship between levels of ERK1/2 activity in the PPT and total number of REM sleep episodes (*F*_(1,28)_ = 207.18; *R*^2^ = 0.88, *p* < 0.001; Figure 5B). These results suggest that the U0126 microinjection-induced reduction in RSHD might have been caused by the reduction in ERK1/2 activity in the PPT.

## Discussion

**Figure 6:**
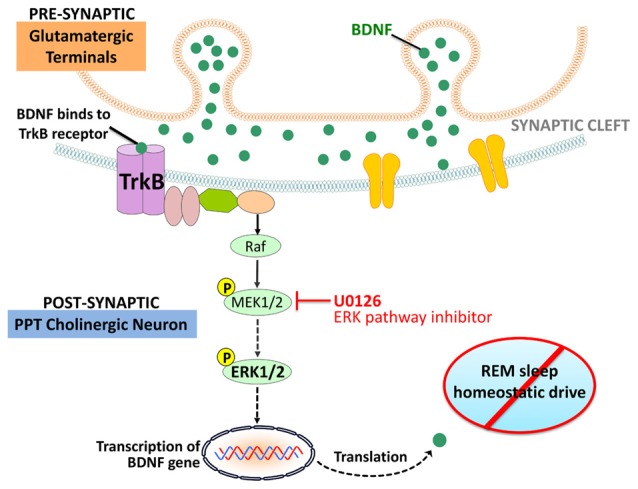
Working model depicting molecular steps in the PPT cholinergic neurons involved in the regulation of RSHD. Arrows: normal activating effects on the target molecule. Dotted arrows: loss of activating effects on the target molecule due to application of U0126 (ERK1/2 activation inhibitor).

The present study highlights the interaction between the BDNF and ERK signaling system in the PPT and its role in the homeostatic regulation of REM sleep. The principal findings of this study are as follows: (1) homeostatic drive for REM sleep increased during short-term selective RSR; (2) during short-term RSR, BDNF protein expression and ERK1/2 phosphorylation and activation increased in the PPT; (3) local application of the ERK1/2 activation inhibitor, U0126, into the PPT, inhibited ERK1/2 phosphorylation and activity; (4) local application of the ERK1/2 activation inhibitor into the PPT suppressed short-term selective RSR-induced increases in RSHD and BDNF expression; and (5) after local application of ERK1/2 activation inhibitor in the PPT and selective RSR, the amounts of BDNF expression in the PPT and RSHD of individual animals were positively correlated with the levels of ERK1/2 activity. These results provide evidence suggesting that activation of the intracellular ERK1/2 signaling system in the PPT is an important molecular step for the development of RSHD (Figure 6).

Consistent with our previous study, short-term selective RSR resulted in an escalation of RSHD and increased expression of BDNF protein in the PPT (Datta et al., 2015). The results presented here provide evidence to suggest that suppression of BDNF protein expression in the PPT further suppresses selective RSR-induced increases in RSHD. These results also suggest that this short-term RSR-induced RSHD may have been developed by an increased expression of BDNF protein in the PPT. This potential causal relationship between BDNF protein expression in the PPT and RSHD, requires future testing and validation by studies examining the effects of pharmacologically active agonists and/or antagonists of BDNF.

ERK1/2 are expressed in the central nervous system (CNS) and are vital components of the neuronal transduction pathways (Thomas and Huganir, 2004: Raman et al., 2007; Gerits et al., 2008). The activation of ERK1/2 is a critical component of the neuronal response and has been shown to be involved in neuronal maturation and survival, synaptic plasticity, and learning and memory (Kornhauser and Greenberg, 1997; Atkins et al., 1998; Coogan et al., 1999; Dolmetsch et al., 2001; Wu et al., 2001; Paul et al., 2003; Sweatt, 2004; Braithwaite et al., 2006; Paul and Connor, 2010). Interestingly, a number of sleep and memory studies in both animals and humans have shown that learning training increases RSHD during subsequent periods of sleep (Datta, 2010; Groch et al., 2013). For example, emotional and implicit memories are preferentially enhanced across periods of enhanced REM sleep (Datta, 2000; Wagner et al., 2001; Cai et al., 2009; Pace-Schott et al., 2009; Datta and O’Malley, 2013; Groch et al., 2013). Furthermore, the restriction of post-training RSHD impairs consolidation of these memories (Datta et al., 2004; Fu et al., 2007; Spoormaker et al., 2011). The present study highlights the interaction between BDNF and ERK1/2 signaling in the PPT as a causal factor for the development of RSHD. Thus, future studies on learning and memory may want to target this interaction as a potential cellular and molecular mechanism for RSHD-associated memory processing. It has been shown that the levels of phosphorylated ERK1/2 (pERK1/2) and ERK1/2 activity in the PPT are higher in animals with more sleep compared to animals with less sleep (Desarnaud et al., 2011). Additionally, the levels of pERK1/2 and ERK1/2 activity in the PPT were shown to be substantially lower in animals with high wakefulness. Thus, it is reasonable to suggest that the up-regulation of ERK1/2 signaling system in the PPT might be involved in the regulation of consolidated sleep. The results of the present study demonstrated that the inhibition of ERK1/2 activity in the PPT blocked selective RSR-induced increases in RSHD. Therefore, the present study revealed that ERK1/2 activity in the PPT is critical in the homeostatic regulation of REM sleep, thus revealing another important function of ERK1/2 signaling in a neuronal system.

In this study, local application of U0126 into the PPT not only decreased ERK1/2 activity, but also decreased the levels of BDNF protein expression in the PPT. The positive relationship between levels of ERK1/2 activity and BDNF protein in the PPT was highly significant, explaining 89% of the variance in BDNF (*R*^2^ = 0.89). It is known that in the brain, BDNF predominantly binds to the tropomyosin-related kinases B (TrkB) receptor, which activates the ERK1/2 transduction pathway (Segal and Greenberg, 1996; Han and Holtzman, 2000; Huang and Reichardt, 2001; Ying et al., 2002; Kishino and Nakayama, 2003; Alonso et al., 2004; Lu et al., 2004; Mohajerani et al., 2007; Numakawa et al., 2010). It is also been suggested that the TrkB receptors in the PPT participates in the homeostatic regulation of REM sleep (Barnes et al., 2017). It is also known that ERK1/2 signaling increases BDNF release in the brain in a positive feedback loop (Obata et al., 2003; Kelleher et al., 2004; Klann and Dever, 2004). Therefore, it is logical to suggest that in the beginning of selective RSR, increased activation of ERK1/2 signaling in the PPT may have been caused by the increased BDNF protein expression via a positive feedback loop. This sustained positive feedback loop between BDNF and ERK1/2 signaling, may be a causal factor for the development of RSHD (Figure 6).

The results of the present study show that local application of U0126 into the PPT not only decreased RSHD but also decreased NR and increased wakefulness. This result is also consistent with the findings of other studies that have shown sleep deprivation decreases ERK activity and hippocampal and cortical plasticity in rodents (Guan et al., 2004; Ravassard et al., 2009; Park et al., 2012; Ma et al., 2014; Dumoulin et al., 2015; Su et al., 2016). Similarly, studies in *Drosophila* also showed that the regulation of sleep and plasticity involve ERK phosphorylation (Foltenyi et al., 2007; Vanderheyden et al., 2013). Many other studies have also shown that the phosphorylation and activation of ERK increases during sleep (Desarnaud et al., 2011; Soulé et al., 2012; Ikeda et al., 2015; Keshavarzy et al., 2015; Mikhail et al., 2017). The role of ERK in REM sleep regulation is also supported by another study that has shown that TrKB.T1 knockout mice have increased REM sleep time, reduced REM sleep latency, and reduced sleep continuity (Watson et al., 2015). The removal of TrkB.T1 receptors might have reduced its dominant-negative inhibition on TrKB.FL receptors that ultimately increased ERK signaling (Barnes et al., 2017). Based on the results of these studies, as discussed above, it is reasonable to suggest that in the present study inhibition of ERK 1/2 activity and phosphorylation in the PPT might have increased wakefulness by decreasing sleep. Based on this interpretation, one could also argue that the reduction in RSHD in the present study may not be the direct effect of U0126 microinjection-induced inhibition ERK1/2 phosphorylation and activation in the PPT. Rather this is an indirect or non-specific effect of simply the decreased NR and increased wakefulness caused by the decreased ERK 1/2 phosphorylation and activation. However, we believe that it is a highly unlikely possibility, because, in the present study microinjections of U0126 into the PPT reduced only about 30% of their total NR and 20%–30% of baseline NR is sufficient to preserve normal RSHD (Trachsel et al., 1991; Ocampo-Garcés et al., 2000). None-the-less, we acknowledge that a future study is needed to determine whether the effects of U0126 on RSHD and sleep-wake activity are due to directly modulating the PPT or whether the effects of U0126 are due to non-specific effects on physiological phenomenon. It is possible that these effects are physiologically driven, thus future studies should examine the non-specific effects of U1026.

In conclusion, the behavioral, pharmacological, physiological and molecular data reported here demonstrate, for the first time, that a positive interaction between BDNF protein expression and ERK1/2 signaling in the PPT is an important cellular and molecular event for the development of RSHD. The current data provides a novel perspective on modulatory effects of PPT intracellular signaling activity and BDNF expression in the homeostatic regulation of REM sleep. These results also suggest that the REM sleep associated neuronal plasticity and memory processing may involve the interaction of BDNF and ERK 1/2 signaling in the PPT.

## Author Contributions

SD: designed the study, performed experiments, analyzed the data, and wrote the manuscript. MDO: analyzed the data and co-wrote the manuscript.

## Conflict of Interest Statement

The authors declare that the research was conducted in the absence of any commercial or financial relationships that could be construed as a potential conflict of interest.
